# Walking the fine line: Self-reported reasons for substance use in persons with severe mental illness

**DOI:** 10.3402/qhw.v8i0.21968

**Published:** 2013-12-20

**Authors:** Henning Pettersen, Torleif Ruud, Edle Ravndal, Anne Landheim

**Affiliations:** 1 National Centre for Dual Diagnosis, Innlandet Hospital Trust, Brumunddal, Norway; 2 SERAF – Norwegian Centre for Addiction Research, University of Oslo, Oslo, Norway; 3 Division Mental Health Services, Akershus University Hospital, Lørenskog, Norway; 4 Institute of Clinical Medicine, University of Oslo, Oslo, Norway

**Keywords:** Psychotic disorder, models of comorbidity, self-medication, assertive community treatment, patient experiences, qualitative study

## Abstract

Many theoretical models have been proposed to explain the relationship between severe mental illness (SMI) and substance use. Because many of these are contradictory quantitative American studies, a qualitative, exploratory study of a Scandinavian sample may offer a new perspective. The aim of the study is to explore reasons for substance use through analysis of the participants’ experiences. A qualitative study with semistructured interviews was used. Purposeful sampling (*N*=11) of patients with substance use disorder (SUD) and SMI, who were included in assertive community treatment teams, was completed. Inclusion criteria are increased quality of life or increased general functioning, and decreased substance use, after a minimum of 12 months in treatment. Reasons given for using substances were categorized as (a) controlling the symptoms of mental illness, (b) counteracting medication side effects, or (c) balancing the ambiguity. The conclusion is that the study findings mainly support secondary substance use models in explaining the comorbidity of SMI and substance use. However, there is some support for the traditional self-medication hypothesis (SMH), iatrogenic vulnerability, and the supersensitivity model. This may be because the majority of the study participants reported having a mental illness with subsequent substance use. The expressed ambivalence to substance use also lends some support to bidirectional models.

Both epidemiological (Kessler et al., 1996; Regier et al., 1990) and clinical studies (Duke, Pantelis, & Barnes, 1994; Ziedonis & Trudeau, 1997) indicate that persons with severe mental illness (SMI) are more likely to have a substance use disorder (SUD) than others. It is likely that these persons benefit from traditional treatment to a lesser degree than others and show a poorer prognosis concerning both mental illness and SUD and that the substance use has implications for both the treatment and course of mental illness (Blanchard, Brown, Horan, & Sherwood, 2000; Buckley, 2006; Dixon, Haas, Weiden, Sweeney, & Frances, 1990). Individuals with schizophrenia who use drugs have a higher risk of hospitalization than those who abstain from drugs; they have a higher risk of suicide and are more prone to homelessness (Cohen, Test, & Brown, 1990; Drake et al., 1990). The most commonly used substances for persons with a diagnosis of schizophrenia or other psychotic disorders are cannabis, alcohol, and stimulants (Atakan, 2008; Dixon et al., 1990; Koskinen, Löhönen, Koponen, Isohanni, & Miettunen, 2010).

Qualitative studies show that individuals diagnosed with SMI experience substance use as having a negative impact on mental illness (Charles & Weaver, 2010; Cruce, Öjehagen, & Nordström, 2008). However, there are other studies where participants held strong beliefs on the use of substances to self-medicate, to relax, and as a break from illness (Asher & Gask 2010; Francoeur & Baker, 2010). Further along this line, the argument is that the subjective expressions of individuals with schizophrenia to use drugs seem to be that they perceive themselves as self-medicating symptoms (Dixon et al., 1990). A recent mixed-method study shows that relief of dysphoria was the most frequently endorsed reason for substance use. Furthermore, they found that few participants reported alcohol and cannabis use as means to alleviate psychotic symptoms or to medicate side effects (Thornton, Baker, Johnson, Kay-Lambkin, & Lewin, 2012).

Several types of theoretical models attempt to explain the increased substance use by individuals with psychotic disorders: *Common factor models* propose that one or more factors independently increase the risk of both mental illness and substance use. *Bidirectional models* hypothesize that either disorder can increase vulnerability to the other disorder. *Secondary psychopathology models* argue that substance use causes psychiatric disturbances that would otherwise not have developed. Finally, *secondary substance use models* claim that the high rates of comorbidity are the consequence of primary SMI leading to SUD (Moggi, 2005; Mueser, Drake, & Wallach, 1998). With regard to SMI, most empirical research is found on the latter model, although there is a considerable amount of research on cannabis use precipitating mental illness.

As one of the secondary substance use models, the traditional *self-medication hypothesis* (SMH) assumes that specific substances are used to alleviate specific symptoms of the psychosis and to gain relief from negative affect and stress (Khantzian, 1985, 1997). The hypothesis has been referred to in several studies (Bizzarri et al., 2009; Phillips & Johnson, 2001; Schneier & Siris, 1987). Although it has little support in clinical research (Mueser et al., 1998), several studies on clients’ self-reports point to the relief of dysphoria as a motivational factor for substance use (Addington & Duchak, 1997; Dixon et al., 1990; Spencer, Castle, & Michie, 2002). By focusing on emotional states rather than symptoms, and less on specific substances to address specific symptoms, the general version of the SMH is more in line with the *alleviation-of-dysphoria model*.

Often mentioned in relation to the SMH is the reasoning that use of antipsychotic medication can cause a state of vulnerability that causes or legi-timizes the use of non-prescribed drugs (Khantzian, 1997). Some studies support the theory of iatrogenic vulnerability (Costain, 2008; Duke et al., 1994; Schneier & Siris, 1987; Spencer et al., 2002). A literature review on self-reported reasons for substance use showed the side effects of medication to be an important motivational factor for substance use in several of the studies (Gregg, Barrowclough, & Haddock, 2007).

The *supersensitivity model* claims that increased biological vulnerability to the effects of substance use can explain some of the comorbidity of these disorders (Moggi, 2005; Mueser et al., 1998). Supportive of the model are findings that individuals with dual disorders seem to use lower quantities of substances than those with primary SUD (Lehman, Myers, Corty, & Thompson, 1994) and that small amounts of substances are likely to induce psychiatric symptoms among clients with SMI (Drake, Osher, & Wallach, 1989). Few clients with SMI are able to sustain moderate substance use over time without experiencing negative symptoms (Drake & Wallach, 1993). Among the different etiological models of secondary substance use, research seems to provide the strongest support for the supersensitivity model and partly the alleviation-of-dysphoria model (Mueser et al., 1998). However, many of the theoretical models that try to explain the relationship between SMI and substance use are contradictory, quantitative studies based on US samples.

Participants for our study were selected from five of the assertive community treatment (ACT) teams established in Norway in 2007–2010. A qualitative study from a Norwegian setting may contribute by exploring how the persons themselves describe the reasons for their use of substances. The main discussion in the article views the participants’ experiences of substance use in light of different theoretical models that seek to explain the comorbidity. Few studies describe how persons with SMI explain their use of substances. This article seeks to fill a gap in the contemporary literature. The aim of the study is to explore reasons for substance use through analysis of the participants’ experiences.

## Methods

This study used a descriptive and explorative design, aiming to create knowledge about individuals’ experienced life and subjective meanings. The objective was to describe how the participants’ substance use is experienced and reflected upon in order to shed light on the reasons for substance use as a subjective phenomenon.

### Recruitment and ethical aspects

A strategy of criterion-based, purposeful sampling was used to recruit patients from five ACT teams throughout Norway (Patton, 2002). Contact was first established by telephone with the team leaders of the five teams that had the most experience as ACT teams and that had included the most patients. This contact was followed by an e-mail with written information explaining the purpose of the study. The e-mail requested permission for the first author to interview patients who met the inclusion criteria for the study: persons with simultaneous SUD and SMI who were included in ACT teams, and who had increased quality of life or better functioning, or a decrease in substance use (as defined by both the patient and the team), after a minimum of 12 months of treatment. Current substance use was assessed using the Alcohol Use Identification Test (AUDIT) and Drug Use Disorders Identification Test (DUDIT) when the patient joined the ACT team, and problematic substance use was considered meeting the inclusion criteria of an SUD.

The team leaders recruited the participants by asking the rest of the team if they had patients who met the inclusion criteria for the study. We do not know exactly how many patients were asked to participate and how many refused to take part in the study. However, our impression was that most of those who were asked did agree. Two of the teams that did not contribute participants to the study had recruited three more patients, but they were not included because the sample size was already sufficient. The team leaders also made appointments for the interviews.

The relationship with some of the participants was rather fragile in the sense that revealing too many difficulties and private issues during the interviews could lead to later feelings of distress. This was expressed by some of the participants as well as by some of the team leaders. Therefore, it was arranged that the first author, who performed all the interviews for this study, would report back to the team on the progress of all patients after the second interview without disclosing the content of the interviews.

The study was approved by the Regional Committee for Medical and Health Research Ethics, South-East Region (no. 1196, 2010), before patients were invited to participate. Each individual gave written informed consent to participate in the study.

### Participants

Eleven patients (nine men and two women) met the inclusion criteria. At the time of the first interview, the age range was 27–63 (mean age = 39 years). Most participants had a long history of SMI, with subsequent substance use. Only one participant was unsure whether his substance use preceded his psychotic disorder or vice versa. The majority of the participants had a diagnosis of schizophrenia, but some individuals had bipolar disorder and unspecified psychosis. All participants had a history of using substances, mainly amphetamine and cannabis, and, to a lesser extent, alcohol and prescription drugs. Four participants had quit substance use by the time of the interviews. One of those had also injected heroin. The seven participants who were still using substances mainly reported using amphetamine, cannabis, and alcohol, in nearly equal amounts, and mostly in a mixture of use. One reported using only prescription drugs (e.g., benzodiazepines and codeine). Few of them seemed to use substances on a daily basis. Most typical was to take some substances 3–4 times a week, often in connection with lapses in their mental illness. The majority of the participants had originally been medicated with antipsychotics to treat their mental illness, but only one of them was on forced medication.

The majority of the participants lived alone, and most of them were in rented flats owned by the municipality. Only two of the participants were employed part-time in government-subsidized work, but some were about to start full-time work or had plans to complete a certificate of apprenticeship. Three had unfinished culinary qualifications. All of the participants had experienced different treatment settings due to both their mental illness and their substance use before they were included in an ACT team. At the time of the first interview, the length of treatment in the team ranged from 14 to 36 months (mean treatment time = 22 months).

### Interviews

The first individual interviews were completed between August and November 2011, and the second interviews, with nine of the 11 participants, were completed between February and June 2012. The interval between the first and second interviews was 5–8 months. One participant was considered by his therapist to be unable to complete a second interview because of a relapse, and one participant did not meet at the scheduled time of the interview. A total of 20 interviews were conducted; 11 took place in a meeting room frequently used by the ACT team, six were performed in the participants’ homes, and three took place in an in-patient setting. The duration of each interview ranged from 45 to 75 minutes, and most interviews were recorded with a digital sound recorder. One of the participants objected to the use of recording devices during the interview, so data from this interview were recorded in written notes. Most participants required one or two breaks during the interview. Most interviews were on a one-on-one basis, but on two occasions a nurse or therapist accompanied the participant for safety or support reasons. On the first occasion, a clinical nurse was present during the interview due to safety reasons. The participant had on some occasions displayed aggressive behavior. The presence of the nurse seemed not to influence what was talked about, and the interviewer felt confident in his presence. On the other occasion, a clinical psychologist attended the interview for supportive reasons on request from the participant. The participant described a close relationship to the psychologist and addressed him twice during the interview. But overall, this interview also progressed so that significant information was passed on. The second interview with both participants was conducted without assistance from the therapists.

An interview guide with specified topics was used to focus on the relevant experiences of the participants, including reasons for the use of the substance, positive and negative aspects of substance use, and how substance use influenced their mental illness. Probing questions were used to further explore the issues that were brought up. Two of the participants did not accept, or seemed unaware of, their psychiatric condition. Hence, asking how substance use influenced their mental illness was difficult. But both of them could express how substance use influenced their mental state—concerning, for example, depressive thoughts, anxiety, or well-being—without agreeing on their diagnosis of schizophrenia. In most instances, each interview was transcribed, and a transcription memo taken, before interviewing the next participant.

The reasons for doing a second interview were to explore important issues not covered during the first encounter, clarify information given in the first interview, and have the opportunity to more thoroughly examine specific topics. The follow-up interviews provided a longitudinal dimension. It has been stated that reporting to respondents what they have said in a previous interview elicits better data (Farrall, 2006). However, having several months between the interviews was disadvantageous in that most participants found it hard to recall what they talked about in the first interview. To ease the memory, each participant read a transcript summary from the first interview or was presented with main points from the transcription of the first interview as an aid to call to mind important issues from the first encounter. Their feedback formed the basis of the subsequent theme development in the second interview and served as a means of internal validation (Aronson, 1994). Furthermore, none of the participants reconsidered or contradicted what they had brought up during the first interview.

### Analysis

The interview transcripts were analyzed using systematic text condensation (Malterud, 2012), a pragmatic approach inspired by phenomenological psychology (Giorgi, 2009). This includes a stepwise procedure that aims to identify recurring initial codes and themes relevant to the purpose of the study. The method is recommended for descriptive and explorative analyses of a phenomenon in reports from different participants. Its use is also suggested when developing new descriptions of a phenomenon. An inductive approach was intended in the sense that the identified themes were strongly linked to the data themselves (Charmaz, 2006).

The first author carried out the data collection and performed most of the analysis. By having the ACT teams recruiting patients for the study, the first author (interviewer) knew nothing more of the participants than their gender, their psychiatric diagnosis, and that they were engaging in substance use when included in the team. The first author, trained as a clinical nurse and with a master's degree in health promotion, focused on factors influencing progress and well-being. The interview transcripts were read with an open mind, as a means to bracket the researcher's preconceptions and with focus on what the participants conveyed.

Notes taken after each interview made it easier in the consecutive interviews to remind the interviewer to ask questions and give prompts that were more tailored to each participant, be prepared for the physical environments, and reserve an appropriate amount of time and prepare for convenient breaks to conduct each interview.

To sort out and organize the interview data, the software program NVivo 10 (QSR International Pty Ltd., 2012) was utilized in the theme development after the initial codes were identified. Initially, the interview transcripts were read through to search for expressions viewed as important aspects contributing to improvement. A total of 115 expressions (initial codes) were identified. Further searches for familiarity and diversity among the initial codes resulted in six overarching themes covering the whole data set. The main theme, *Different aspects of substance use*, was selected for analysis and covered all of the expressions from the participants concerning substance use. Because of the large number of meaning units within this main theme, it was split into *Substance use as a coping strategy* and *Experiences of abstaining from substance use*. The former theme was selected for further analysis. A total of 43 meaning units, consisting of sentences or paragraphs from the transcripts, were identified. The meaning units were organized into three subthemes, and a text of condensed meaning was constructed for each one. The last phase consisted of summarizing the meaning of the content in a new description.

Most of the initial coding and initial theme development were done by HP, but AL and ER contributed significantly in the identification of codes in the initial phase, and through the later theme development.

## Results

The findings emphasize the different reasons that participants gave for their substance use, and they are classified in relation to different models to explain the co-occurrence of SMI and substance use. Both former and present users contributed to the findings. Below, the participants’ expressions are reported and discussed in accordance to the following subthemes: *controlling the symptoms of mental illness*, *counteracting medication side effects*, and *balancing the ambiguity*.

### Controlling the symptoms of mental illness

Although some of the reasoning for substance use was as a way to relax or get high, this was not the main reason for substance use in our study. Instead, the focus was on experiences of emotional states and how these were influenced by substance use. Some of the participants who used, or had used, alcohol regularly explained that the main purpose was to decrease anxiety and depression, and/or to take a break from everything that was difficult. The use of alcohol was viewed as less harmful than the use of illegal substances, while other expressions underscored a preference for cannabis or amphetamines, mentioning alcohol in negative terms and suggesting it caused problems for individuals as well as for society.

Reasons for substance use were seen as both a way to escape unwanted conditions and a way to create a shift of focus. Often-cited reasons were to get a break from experienced difficulties, and this was expressed through the use of different substances. Concepts used to illustrate this were *break*, *interruption*, *sedation*, and *escape*. Some of them had established strategies to implement these timeouts in their everyday life on a predetermined time schedule, while others just did it when it felt right.

One participant (Participant 1, or P1) had used alcohol for many years in a conscious strategy to manage severe distress:

Twenty-four days can pass, then the 12 beers eliminate all the problems, give me a timeout. Being drunk takes me to another place where I'm happy, can start over again and begin on another 24 days. Then, another 12 beers. That's how my life goes. God would thank me for making it through another period. (P1)

Other expressed reasons for using alcohol were to calm down and/or to sleep at night. The use of benzodiazepines was described as creating a general state of calm and daze, even when hearing internal voices. The use of prescribed drugs was not a big issue for those who participated, and their use seemed to be associated with the use of other substances. Cannabis was viewed specifically as a way to cope with the hearing of voices. In most cases, this was mentioned in relation to alleviating states of extremely loud and dominant voices. One participant also explained that cannabis had a more calming effect on the voices than the use of antipsychotic medication. He described how substance use affected his mental illness:

Hash helps me calm my inner voices when they get loud. I feel it's the only medicine that helps. Better to smoke dope than to drink alcohol. Fluanxol can also help me with the voices but not all the time. Dope, on the other hand, affects me deeply. It penetrates into my bones and all around me. The THC [tetrahydrocannabinol] in the dope encases itself around my bones, enhances my senses by making them pure in everyday settings. (P6)

The use of amphetamines seemed to have several functions. Other given reasons were the use of amphetamine to regulate, or even induce, the manic states of a bipolar disorder. The mania under the influence of amphetamines was perceived to be less troublesome than other manic episodes. One participant, who was diagnosed with bipolar disorder, started using substances as an adult and described his strategy of using amphetamines in the following manner:

My manic periods are experienced differently when I'm high on amphetamines compared to when I'm not. Being high makes me feel stronger during my manic phases. Amphetamines help me achieve a manic phase that is similar to a manic phase without amphetamines. The dope helps me identify and learn from the symptoms in a manic phase. If I didn't, then the manic phases would come less often and I would not be able to cope with them [the manic phases] when they come. (P9)

Because controlling and stabilizing efforts are important in coping with his illness, amphetamines become an agent to induce more frequent, but less dramatic, manic episodes. Other given reasons were the calming effects of amphetamines when hearing voices; amphetamines seemed to be more potent than cannabis in this respect.

The urge to have substances was strongly related to the episodes and fluctuations associated with mental illness. The need to take substances seemed to be less urgent when symptoms of the psychotic disorder did not dominate. As one participant pointed out:

I don't need to get high all the time. I feel I need to get high when I get the tiresome fluctuations [swings]. I can also calm myself down with some coffee and a cigarette, without the need of drugs. Things have changed a bit, I have more control now. (P6)

Experiences of ups and downs seem to be the nature of severe mental disorders. Some expressions indicated that the illness developed more or less independently of the substance use. The illness was something that could not be cured, but the substance use could be seen as a strategy to make life worth living.

### Counteracting medication side effects

Amphetamines seemed to be the preferred substance to address the experienced side effects of antipsychotics. Statements about substance use reflected the need to create more “awake” days, as antipsychotic medication imposes a drowsy state and an urge to sleep both day and night.

One participant described himself as a former polydrug user. He reflected on his substance use after being abstinent for several months:

Getting high on amphetamines wasn't so bad. Zyprexa caused my anxiety and made me sleepy. I started using amphetamines to do something about the side effects of my antipsychotic medicines. I remember that I slept so much that all I wanted was a day or a weekend where I was awake. (P3)

Gaining weight is a well-known side effect of these medicines, and some of the reasoning expressed the use of amphetamines as a slimming strategy. In some instances, this extended into its use as a strategy to increase well-being and stimulate the courage to participate in social activities. In comparison, one participant had his own strategy in the sense that he himself made the decision to quit the use of antipsychotics, more or less against advice from his psychiatrist. Being abstinent for the last 2 years after using some amphetamines, but mainly alcohol, to relieve anxiety, he explained:

My medicines caused my weight gains. They suppressed my feeling of fullness. It felt like I was hungry at all times and [so I] ate. After that period, I stopped taking my medication and went from 115 kg to 85 kg. I function better without medication than I did when I was on them. My cravings for alcohol also disappeared. My quality of life is better now after I quit using both alcohol and my medicine. (P4)

Instead of continuing to use alcohol to counter the unwanted effects of the medicines, he stopped using antipsychotics. His strategy had an extensive impact—he did not need either alcohol or amphetamines to manage his daily life.

Expressed reasons for the use of amphetamines seem to imply increased substance use in periods of heavy medication. The rationale expressed for this use implied an attitude of stubbornness in not fully agreeing with the use of medication. In a wider sense, the participants emphasized the need to be in charge of their own lives. To some extent, the medication seemed to create a sort of imbalance, and amphetamine use was claimed to help restore this balance. This reflects the views that amphetamines were considered to be the polar opposite of antipsychotics and that a kind of equilibrium was gained through this strategy.

One participant, who was diagnosed with bipolar disorder, expressed his reasons for using amphetamines:

Yeah, I can envision a life without getting high … a fantastic life without amphetamines. But taking Cisordinol and being dependent on it, feeling blue and emotionally flat … like the last ten years, makes it real hard to quit using amphetamines. (P9)

He had been on medication for several years, but the use of substances (mainly amphetamines) started later in his life. This can be seen as an example of informed choice in the sense that he has experienced SMI both with and without substance use, and he decided on the latter to manage his life.

### Balancing the ambiguity

Reasons given for substance use contained elements of ambivalence or ambiguity. The same substance had different meanings, effects, and consequences for different individuals, and the overall perspective seemed to applaud the positive short-term effects of taking substances but at the same time showed awareness of the adverse long-term consequences. Substance use seemed to create problems but, at the same time, produced better functioning in different arenas. All over, reasons for substance use reflected ambivalence towards which consequences substance use have for daily life.

Some expressions indicated that amphetamines contributed to create energy and activity in daily life, but at the same time led to unpleasant tremors and later deterioration of well-being. One participant described the very effective way that amphetamines helped him to lose weight, but it was too fast. He appreciated the fantastic effects of the substance but, at the same time, feared the consequences:

I lost weight, but using amphetamines now doesn't make me feel any better; I sometimes use hash so I do not become too thin, it is better to lose weight slowly. I can't wait to get to a more stable weight where I feel better about myself. Taking amphetamines makes it easier for me to live. But it is not healthy to be constantly in this situation. (P6)

There were also statements about the impact of substance use on mental illness. Broadly speaking, there seemed to be more worries concerning the amount of substances being used than about the adverse effects inherent in the specific substances. Some expressions on the use of substances to self-medicate unpleasant states and symptoms reflected the risk of a worsening of mental illness and becoming psychotic. One participant commented on how substance use may lead to both well-being and deterioration:

I don't think getting high really affects my mental problems. I feel that I am more functional when I'm high, while at the same time there is a danger of taking too much and ending up in a psychotic state. (P7)

This awareness of the harmful effects of substance use can be transferred to the ambivalent view on antipsychotic medication. This ambivalence was displayed through expressions of the important contribution of antipsychotics to help participants improve, and at the same time complaints about its adverse effects. One of the participants described his experiences of being in active psychosis under the influence of amphetamines as quite different from active psychosis without the substance. He described the risks and benefits associated with amphetamine use in severe states of his illness. Also prevalent were expressions of the negative influence of substance-using environments, even in periods of abstinence. Expressions of ambiguity seemed integrated when weighting the advantages of substance use against the adverse effects. Several of the participants could easily recall the negative influence of the substance-using environment, and they couldn't fully believe the sobriety was going to last.

Despite most of the substances being illegal, and despite all of the negative consequences connected to substance use, substances were being used. Expressions were prevalent of life as a struggle of conflicting emotions with unpredictable outcomes. One description contains experiences of being both abstinent and an active user of substances:

Even though I've had drug-free periods, my life hasn't gotten any better. I am still fighting the same fight. Not that it's any better when I'm on drugs. (P7)

Personal narratives of the impact of substance use on daily life reflect the basic uncertainty that the participants experienced in their lives. Substance use leads to both well-functioning and malfunctioning outcomes for those trying to keep up with the demands of family, therapists, and the greater society while facing SMI symptoms.

## Discussion

The aim of this explorative study was to examine the reasons for substance use through the participants’ experiences. The analysis showed different views on substance use. First, active use could be seen mainly as a means to self-medicate the symptoms of mental illness or to alleviate the adverse effects of antipsychotics. Second, ambivalent use indicated both the experienced positive and negative consequences of different substances.

Reasons for substance use were stated mainly as a strategy for managing difficult emotional states and severe symptoms. What is important in this study is that the same substances served different purposes for different individuals. For instance, amphetamines were used for their calming effect after hearing voices, to compensate for emotional flattening, and as a slimming strategy. All of these views were expressed by individuals with similar psychiatric diagnoses.

Findings from our study can be seen as supportive of the *traditional SMH* (Khantzian, 1985, 1997) because specific substances were used to cope with specific symptoms or emotional states. This is inconsistent with a review of self-report studies showing that substances were used to alleviate states of dysphoria (Gregg et al., 2007), rather than that specific substances were used to handle specific symptoms. Our results also differ from those of two other studies on patients’ perspectives (Charles & Weaver, 2010; Cruce et al., 2008), which found that substance use was considered to have mainly negative effects on SMI. In contrast, reasons given in our study were that, to a large extent, substance use contributes to better management of mental illness (e.g., managing manic episodes, coping with the hearing of voices, calming down the participants, and providing energy). Another crucial finding is that several of the expressions pointed to the importance of taking a break from the challenging facets of *life*. This was done either as a conscious and scheduled strategy, or as a kind of self-help on the basis of experienced dysphoria. However, it is unclear what can be viewed as symptoms, emotional states, and dysphoria (Henwood & Padgett, 2007). The main difference between the findings in the reported studies and ours was that the participants in the reported studies generally had a primary SUD, while participants in our study had several years of SMI with subsequent substance use. This may be critical in explaining the differences between the findings. One important aspect could also be that the majority of participants in our study did not report any negative effects of substance use, probably because few of them ever experienced severe substance use. Another difficulty arises when taking into account that the traditional SMH of Khantzian is based on research on users of heroin and cocaine in an American setting, although his reconsideration of the model (Khantzian, 1997) evolves from diagnoses to emotional states, and he takes into consideration several other substances. The expressions communicated in the current study are consistent with the *traditional SMH*, containing descriptions on how different substances affected difficult emotional states, and indicate that substance use was prolonged despite negative long-term effects. However, the use of different substances to alleviate similar distress among different individuals could also imply that the chosen substance was influenced by availability.

The importance of substance use in coping with the adverse effects of medication was communicated, despite the fact that medication was not a separate issue asked about in the interviews. Iatrogenic vulnerability to antipsychotic medication is one model that attempts to explain the comorbidity of SMI and SUD. The theory is based on the mechanism of antipsychotics, which block some types of dopamine receptors in the brain in order to control psychotic symptoms. This could result in an underactive dopamine reward system and increased vulnerability to substance use (Stahl, 2008). According to Drake, Xie, McHugo, and Green (2000), there is some evidence to support this model, since there is a tendency for clients treated with second-generation antipsychotics to use a smaller amount of substances than those treated with traditional antipsychotics. The rationale is that traditional antipsychotics block more of the dopamine receptors than those developed later. Expressions given in our study indicate that there are quite divergent views on the impact of antipsychotics. Because medication was explored in the context of substance use in this study, it is possible that the adverse effects of medication were focused on. Some of the participants also spoke of antipsychotics as necessary and helpful in stabilizing their lives, but never as a remedy that was purely advantageous. The findings of the current study indicate that substances were used for self-medication and/or to relieve side effects of medication, and, in this respect, the findings oppose findings from other studies concerning the use of alcohol and cannabis (Addington & Duchak, 1997; Thornton et al., 2012). In these studies, participants reported on the use of both alcohol and cannabis as contributing to a worsening of the positive symptoms of psychosis. There are several possible reasons why this was not found in the present study. Participants in the Thornton study (Thornton et al., 2012) were considered to have relatively high functioning and did not go through personal interviews, while participants in the current study were “hard-to-reach” clients in an assertive outreach setting. Addressing the use of amphetamines in the present study may add important information about the reasons for substance use. There are arguments regarding the protective agency of antipsychotics against the neurotoxic effects of amphetamines (Bramness et al., 2012), although the participants’ use of amphetamines counteracts the effects of antipsychotics. According to Bramness et al. (2012), psychosis also seems to be precipitated by a lower dose of amphetamines in individuals with primary psychosis and may be blocked by the use of antipsychotics. No literature was found to shed light on clients’ experiences of using substances when medicated with antipsychotics.

Prominent in the present study were expressions of the importance of taking the right substance in a proper way to minimize adverse effects. In this respect, amphetamines were the most preferred substance. Views on the scope and extent of the use of stimulants are not prominent in the research literature. There are few studies on how individuals with psychotic disorders use and experience the effects of amphetamines (Nolte, Wong, Latchford, Boyle, & Anaenugwu, 2012), although some research indicates that individuals diagnosed with schizophrenia may more readily become psychotic after their use (Lieberman, Kane, & Alvir, 1987). One central aspect of the supersensitivity model is that both genetic and early environmental events interact with later environmental stressors to contribute to psychiatric disorders or to trigger relapses (Mueser et al., 1998). An extension of this argument implies that medications can decrease vulnerability, while substance use can increase it. Participants in our study also point to experiences of going into psychosis when taking too much of a substance. The balance has to be maintained. It seems that after years of experiencing life with SMI, they were aware of their limits. They seemed to use a limited amount of substances, and they had experienced more positive than negative effects. In this respect, the findings support the supersensitivity model.

Some expressions convey that substance use increased during bad periods of the mental illness. In periods of reduced symptoms or emotional distress, the need to take substances was also reduced. This could imply some support to the *bidirectional models*, which claim that mental illness and SUD can have reciprocal influences on one another. In contrast, participants also stated that the mental illness itself seemed to develop more or less independently of the substances being taken. It seemed necessary for the participants in this study to be in control of their substance use. Their experiences of well-being versus distress were made up of a mixture of substance use, medications, and symptoms of mental illness. It could also be argued that *secondary psychopathology models* may explain some of the substance use, such as in the reported episodes in which substance use led to a worsening of symptoms. However, this seems to be of less concern to the participants in our study because most indicated that their mental illness preceded their substance use. The limited amount of substances being used also gives poor support for the latter model.

Furthermore, findings from our study partly contrast previous research on patients’ perspectives. One study (Cruce et al., 2008) found that the desire to use drugs declined when patients were struggling against symptoms of mental illness and that substance use decreased their thinking capacity. Reasons given in our study are contrary. Particularly, the use of cannabis and amphetamines could dampen the hearing of voices and promote clear thoughts. The latter is similar to findings from other studies on cannabis use by individuals diagnosed with schizophrenia (Costain, 2008; Francoeur & Baker, 2010). Substance use in order to have a break from a difficult and monotonous life was stated as a reason in our study concerning the use of alcohol, cannabis, and amphetamine. Similar findings are reported in studies on cannabis use (Costain, 2008; Francoeur & Baker, 2010) and on polydrug use (Asher & Gask, 2010). Findings in the study of Charles and Weaver (2010) indicate that substance use has a negative impact on mental illness and that, among the reasons given for *trying* a substance, the relief of psychotic symptoms was never included. Our study did not examine why the participants initiated their substance use but mainly investigated reasons for why they kept on using. Contrary to the present study, the other studies cited here had samples where a majority of the participants was, or had been, into heavy substance use, and SUD for most of them had preceded the SMI. One important question is whether substance use is viewed in more positive terms when the SMI occurs before the SUD, and therefore as more of a solution than a problem.

### Clinical implications

With regard to self-medication, clinicians should be aware of clients using substances to relieve symptoms of mental illness. Specific substances can be used to counteract both positive and negative symptoms of a psychotic disorder. Established procedures are required in assessments to ask about substance use and the function that the substances have. Clinicians need to motivate the user/patient to reduce the use of substances, and the patients need to get treatment for anxiety and depression. How to live with voice hearing is also important in treatment. In general, to use evidence-based psycho-educative approaches and integrated dual disorder treatment (Mueser, Noordsy, Drake, & Fox, 2003) is essential. Assessing substance use, and how and why clients use substances, is just as important as which substances they use. It is possible that less severe use, such as shorter durations and lower amounts, may be of importance in understanding the relationship between substance use and psychotic disorders. Assessments should also take into account whether substance use has preceded the mental illness or vice versa, as this seems to influence clients’ perception of their substance use. When treating clients with antipsychotics, clinicians should carefully consider the adverse effects of the medications. Some individuals with SMI seem to have a better life when using substances than when abstaining from them. In some cases, this may mean working in collaboration with the clients to minimize rather than eliminate substance use. Clinicians should expand their awareness to meet such challenges.

### Limitations

This is an exploratory study, and the interpretations of the data should be considered within the context of qualitative research. The fact that the study sample consists mainly of individuals with substance use secondary to SMI may limit the potential to explain the comorbidity in terms of etiological models outside secondary substance use models.

Persons with less severe SUD may idealize their substance use and, retrospectively, report fewer negative aspects. The findings are partly based on the participants’ memories of former substance use, and could therefore be susceptible to bias. However, to explore reasons for substance use as a subjective phenomenon involves focus on the meaning the participants give their substance use. The reports on the use of both legal and illegal substances rely on the openness and honesty of the participants.

## Conclusion

This qualitative study, which focused predominantly on participants with primary SMI, sheds light on the reasons for substance use by individuals with SMI in an assertive outreach setting. Substance use was experienced as having a mostly positive effect on symptoms of mental illness, when substances were taken at the right time and in a reasonable amount. The findings mainly support the *traditional SMH*, but they also partly support the *explanatory model of supersensitivity* and *iatrogenic vulnerability to antipsychotics*. *Bidirectional models* could also explain some of the participants’ ambivalence regarding their substance use. Further research may explore how clients diagnosed with schizophrenia and bipolar disorder experience substance use in general, and how they use substances to alleviate the adverse effects of antipsychotic medication. Overall, more research is needed of SUD, both primary and secondary to SMI.
